# Asynchronous electric field visualization using an integrated multichannel electro-optic probe

**DOI:** 10.1038/s41598-020-73538-7

**Published:** 2020-10-05

**Authors:** Shintaro Hisatake, Junpei Kamada, Yuya Asano, Hirohisa Uchida, Makoto Tojo, Yoichi Oikawa, Kunio Miyaji

**Affiliations:** 1 Department of Electrical, Electronic and Computer Engineering, Gifu University, Gifu, 501-1193 Japan; 2 Arkray Inc., Kyoto, 602-0008 Japan; 3 Think-Lands Co., Ltd., Yokohama, 230-0046 Japan

**Keywords:** Electrical and electronic engineering, Imaging and sensing

## Abstract

The higher the frequency, the more complex the scattering, diffraction, multiple reflection, and interference that occur in practical applications such as radar-installed vehicles and transmitter-installed mobile modules, etc. Near-field measurement in “real situations” is important for not only investigating the origin of unpredictable field distortions but also maximizing the system performance by optimal placement of antennas, modules, etc. Here, as an alternative to the previous vector-network-analyzer-based measurement, we propose a new asynchronous approach that visualizes the amplitude and phase distributions of electric near-fields three-dimensionally without placing a reference probe at a fixed point or plugging a cable to the RF source to be measured. We demonstrate the visualization of a frequency-modulated continuous wave (FMCW) signal (24 GHz ± 40 MHz, modulation cycle: 2.5 ms), and show that the measured radiation patterns of a standard horn antenna agree well with the simulation results. We also demonstrate a proof-of-concept experiment that imitates a realistic situation of a bumper installed vehicle to show how the bumper alters the radiation patterns of the FMCW radar signal. The technique is based on photonics and enables measuring in the microwave to millimeter-wave range.

## Introduction

A high-quality beam pattern is critical for millimeter-wave and terahertz (THz)-wave applications such as highly precise and reliable radar detection in autonomous vehicles^1–5^ and high-data-rate wireless communications^6–10^. Beam forming and beam steering^11–13^ are key technologies used in such applications, while many types of array antennas have been developed to implement them^14–16^. Array antennas operating in high-frequency regions are expected to be integrated with peripheral circuits^17–21^ in the near future, which could make near-field measurements to inspect individual antenna components with no antenna terminals to become necessary. The final beam quality, which has a significant effect on the overall system performance, is strongly affected by the surrounding electromagnetic environment. In particular, the higher the frequency, the more complex the scattering, diffraction, multiple reflection, and interference that occur in real situations such as in radar-installed vehicles, transmitter-installed mobile devices, etc., will be^22–25^. In high-frequency regions, it is almost impossible to reflect the real electromagnetic environment such as a bumper, vehicle body, antenna integrated circuit board etc., in the simulation model, with high fidelity, because of the short wavelength^26^. Therefore, in such high-frequency regions, near-field measurements in “real situations” are important for not only investigating the origin of unpredictable field distortions but also maximizing the system performance by optimal placement of antennas, radar modules, wireless transmitters, etc. Such a near-field measurement tool may be indispensable in future autonomous vehicles to periodically inspect the installed millimeter-wave radar radiations, in order to ensure system reliability.

**Figure 1:**
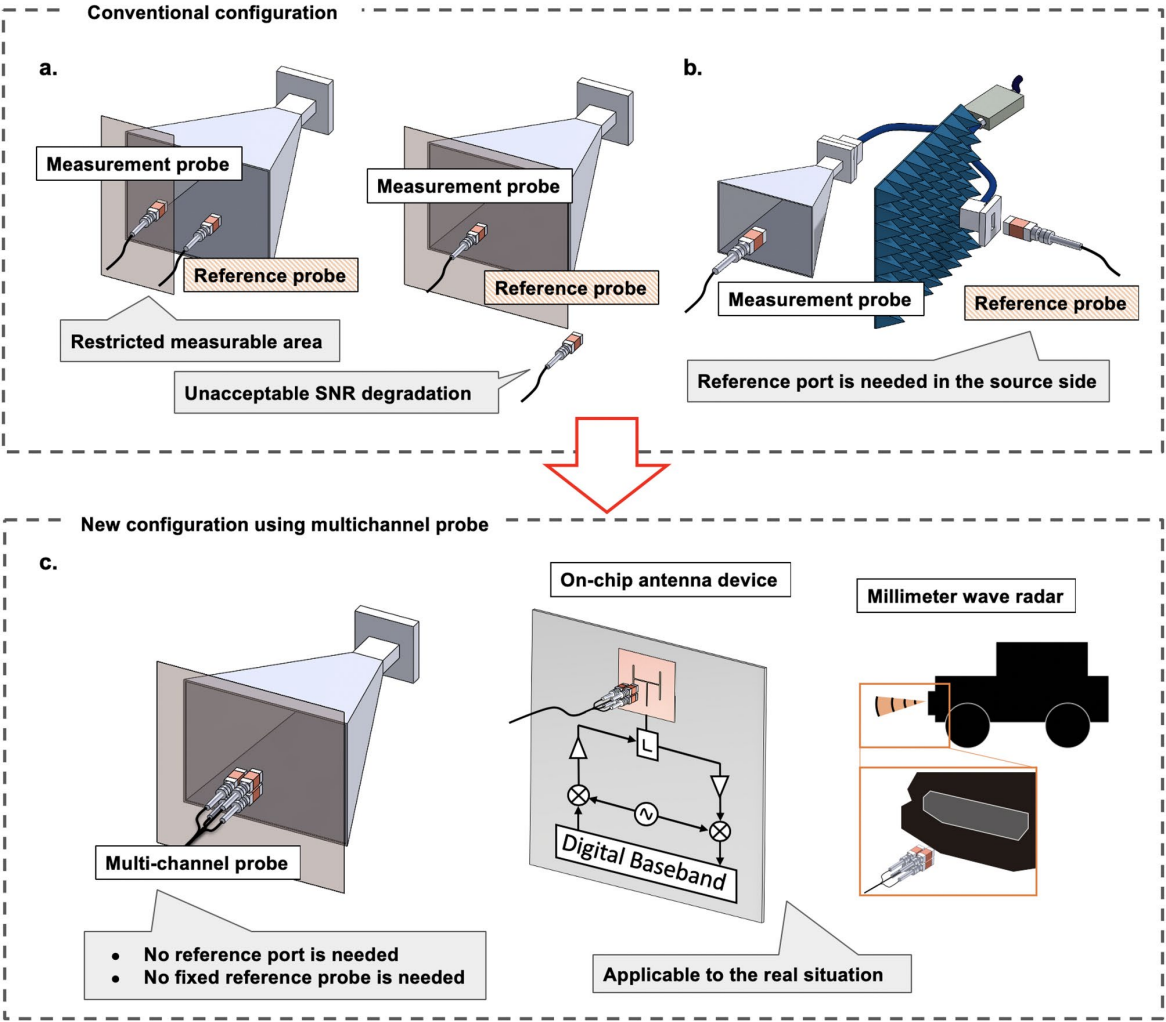
Schematic of the asynchronous measurement technique. (**a**, **b**) Two individual probes (single-channel probe and reference probe) are used in the conventional technique. The reference probe placed on the measurement plane restricts the moveable area of the measurement probe. To increase the measurable area, the reference probe should be set at the edge of the measurement plane; however, this generally results in unacceptable signal-to-noise ratio (SNR) degradation of the reference probe. Therefore, a reference port is required on the source side. (**c**) One integrated multichannel electro-optic (EO) probe is used for the near-field measurements in the new technique, in which no fixed reference probe or reference port is needed.

Conventionally, a reference radio frequency (RF) signal from a measurement setup, such as a vector network analyzer (VNA), is fed to the antenna under test to obtain the near-field phase-distribution measurements^20,27–31^. However, such a measurement system is not suitable for the real situations mentioned above, as it requires cables to be plugged to the device under test (DUT). An asynchronous measurement technique has been proposed as an alternative new technique to map the phase distributions without supplying a reference signal to the DUT^32^, where the electro-optic (EO) probes are used instead of an open-ended waveguide probe. However, this conventional technique also employs a reference probe fixed at a specific point. One option is to set the reference probe at the measurement plane, as shown in Fig. 1a. However, this option is not feasible in a real scenario because of the mechanical interferences that restrict the movable area of the measuring probe or lead to unacceptable signal-to-noise ratio (SNR) degradation of the reference probe. Another option is to set the reference probe at the reference port, as shown in Fig. 1b. Although this configuration still does not require a VNA and is applicable to the self-oscillating sources as validated in^32^, it requires a reference port. Therefore, it is necessary to divide the RF signal to probe the reference signal. However, in most cases (e.g., radar systems), there are no reference terminals (antenna terminals). The necessity of a fixed reference probe for the asynchronous measurements strongly limits the measurement target and scene. To solve this problem, in this work, we propose and demonstrate a new asynchronous measurement technique that uses an integrated multichannel EO probe, as shown in Fig. 1c. The phase distribution is retrieved from the spatial derivatives measured by each three-dimensionally adjacent sensor in the probe. First, we show the near-field visualization of a frequency-modulated continuous wave (FMCW) signal (24 GHz ± 40 MHz with a 2.5 ms period) and compare the far-field pattern calculated from the measured near-field, with the finite integration technique (FIT) simulation results, in order to verify the accuracy of the measurements. Note that the simplest electromagnetic scenario, that is the electromagnetic wave is radiated from the horn antenna, is selected for the validation because more complex electromagnetic scenarios can degrade the accuracy of the simulation. Subsequently, we show in the proof-of-concept experiment, which imitates the realistic situation of a bumper installed vehicle, that the proposed technique can reveal the radiation pattern degradation of the FMCW signal due to the car bumper.

### Principle

**Figure 2:**
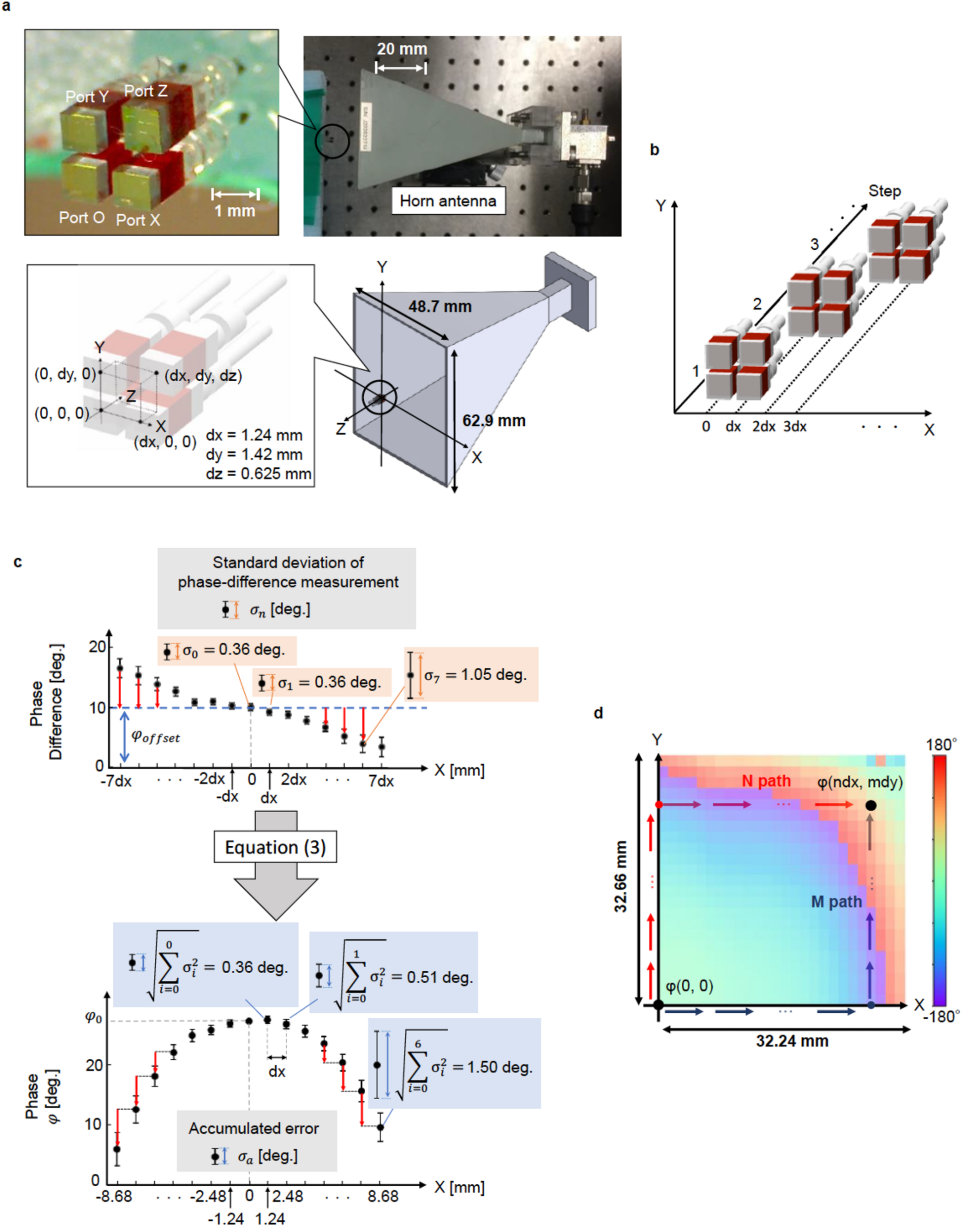
Integrated multichannel probe and measurement method. (**a**) Integrated multichannel probe. The electro-optic (EO) crystal size is 1 mm^3^. dx = 1.24 mm, dy = 1.42 mm, and dz = 0.625 mm. (**b**) Procedure for 1D measurement in the X direction. (**c**) Measured phase difference distribution and retrieved phase distribution. The blue dotted line indicates the calibrated offset phase, $\varphi_{offset}$. (**d**) Retrieved 2D phase distribution.

Figure 2a shows a photograph of the integrated multichannel probe, which consists of four EO sensors. Each EO sensor consists of a high-reflection (HR) mirror, EO crystal, and graded-index (GRIN) lens attached to the polarization-maintaining (PM) optical fiber (see Fig. S1 in Supplementary). A 4-N, N-dimethylamino-4′-N’- methyl-stilbazolium-tosylate (DAST) crystal is used as the EO crystal. The four sensors are placed with spatial separations of dx, dy, and dz, as shown in Fig. 2a. As described later, our system measures the differences between the phases probed by each EO sensor, which correspond to the spatial phase derivatives. We define the difference between the phases measured by port O and port X, port O and port Y, and port O and port Z as $\Delta \varphi_{ox} \left( {x,y,z} \right)$, $\Delta \varphi_{oy} \left( {x,y,z} \right)$, and $\Delta \varphi_{oz} \left( {x,y,z} \right)$, respectively. Here, $\left( {x,y,z} \right)$ is the position of port O.

1 $$ \Delta \varphi_{ox} \left( {x,y,z} \right) = \varphi \left( {x + dx,y,z} \right) - \varphi \left( {x,y,z} \right) $$

where $\varphi \left( {x,y,z} \right)$ is the three-dimensional (3D) phase distribution of the electric field to be retrieved. As shown in Fig. 2b, by moving the integrated multichannel probe in the X direction by a step of dx and measuring the phase difference of $\Delta \varphi_{ox} \left( {x,y,z} \right)$, $\Delta \varphi_{ox} \left( {x + dx,y,z} \right)$, $\Delta \varphi_{ox} \left( {x + 2dx,y,z} \right)$, … repeatedly, the discrete one-dimensional (1D) phase-difference distribution in the X direction can be mapped. In a similar manner, the distribution of the phase differences in the Y direction can also be mapped. In contrast, port Z is placed obliquely behind port O, as shown in Fig. 2a; therefore, the phase difference between the points $\left( {x,y,z} \right)$ and $\left( {x,y,z + dz} \right)$, which is given by $\Delta \varphi_{zdz} \left( {x,y,z} \right) = \varphi \left( {x,y,z + dz} \right) - \varphi \left( {x,y,z} \right)$ can be calculated as

2 $$ \Delta \varphi_{zdz} \left( {x,y,z} \right) = \Delta \varphi_{oz} \left( {x,y,z} \right) - \Delta \varphi_{ox} \left( {x,y,z + dz} \right) - \Delta \varphi_{oy} \left( {x + dx,y,z + dz} \right) $$

Figure 2c shows the results of the measured 1D distributions of the phase differences of a 24 GHz signal on the X-axis and the retrieved 1D phase distributions. As shown here, assuming that the phase at the reference point of $\left( {x,y,z} \right) = \left( {0, 0, 0} \right)$ is $\varphi_{0}$, the phase at an arbitrary point $\left( {x,y,z} \right) = \left( {ndx, 0, 0} \right)$ can be obtained by repeatedly adding the phase differences, expressed by the following equation,

3 $$ \varphi \left( {ndx, 0, 0} \right) = \varphi_{0} + \mathop \sum \limits_{i = 0}^{n - 1} \left\{ {\Delta \varphi_{ox} \left( {idx, 0, 0} \right) - \varphi_{offset} + \varphi_{noise} \left( {idx, 0, 0} \right)} \right\} = \varphi_{0} + \mathop \sum \limits_{i = 0}^{n - 1} \left\{ {\Delta \varphi_{ox} \left( {idx, 0, 0} \right) - \varphi_{offset} } \right\} + \mathop \sum \limits_{i = 0}^{n - 1} \left\{ { \varphi_{noise} \left( {idx, 0, 0} \right) } \right\} $$

where $\varphi_{noise} \left( {idx, 0,{ }0} \right)$ is the noise in the phase-difference measurements, $n$ is an integer, and $\varphi_{offset}$ is the offset phase, which is dependent on the measurement system and which can, therefore, be calibrated in advance. In our case, the phase noise is random noise and is limited by the SNR of the amplitude measurement^32^. The measured standard deviations of the phase-difference measurement, $\sigma_{n} \left( {ndx, 0, 0} \right)$, are shown in Fig. 2c. In our retrieval algorithm, these phase noises are accumulated along the integration path. The resultant standard deviation of the retrieved phase distribution shown in Fig. 2c can be calculated as

4 $$  \sigma_{a} \left( {ndx, 0, 0} \right) = \sqrt {\mathop \sum \limits_{i = 0}^{n - 1} \sigma_{i}^{2} \left( {idx, 0 ,0} \right)} $$

To minimize this error-accumulation effect, the reference point is set at the point where the SNR is the maximum.

The 3D discrete phase distribution can be retrieved from the 3D distributions of the phase differences ($\Delta \varphi_{ox} \left( {x,y,z} \right)$, $\Delta \varphi_{oy} \left( {x,y,z} \right)$, and $\Delta \varphi_{zdz} \left( {x,y,z} \right)$). Figure 2d shows the two-dimensional (2D) phase distributions retrieved from the measured phase-difference distributions. As shown in Fig. 2d, the phase at any discrete point can be retrieved by adding the phase differences along any integration path. It is assumed that $\varphi_{offset\_x}$, $\varphi_{offset\_y}$, and $\varphi_{offset\_z}$ are offset phases dependent on the measurement systems of port O–port X, port O–port Y, and port O–port Z, respectively. In a certain integration path, first, the phase difference is added repeatedly in the X direction. Then, it is added repeatedly in the Y direction, and finally, in the Z direction. The spatially discrete phase distribution $\varphi \left( {ndx, mdy, ldz} \right)$ can be expressed by the following equation,

5 $$ \varphi \left( {ndx, mdy, ldz} \right) = \varphi_{0} + \mathop \sum \limits_{i = 0}^{n - 1} \left\{ {\Delta \varphi_{ox} \left( {idx, 0, 0} \right) - \varphi_{offset\_x} + \varphi_{noise\_x} \left( {idx, 0, 0} \right)} \right\} + \mathop \sum \limits_{j = 0}^{m - 1} \left\{ {\Delta \varphi_{oy} \left( {ndx, jdy, 0} \right) - \varphi_{offset\_y} + \varphi_{noise\_y} \left( {ndx, jdy, 0} \right)} \right\} + \mathop \sum \limits_{k = 0}^{l - 1} \left\{ {\Delta \varphi_{zdz} \left( {ndx, mdy, kdz} \right) - \varphi_{offset\_z} + \varphi_{noise\_z} \left( {ndx, mdy, kdz} \right)} \right\} $$

where $n$, $m$, and $l$ are integers. Here, we retrieve the phase distribution along the N path and M path and average them to reduce the phase-noise accumulation effect. The phase distributions retrieved along the N and M paths shown in Fig. 2d are expressed by the following equations.

6 $$ \varphi \left( {ndx, mdy, 0} \right)_{N} = \varphi_{0} + \mathop \sum \limits_{i = 0}^{n - 1} \left\{ {\Delta \varphi_{ox} \left( {idx, mdy, 0} \right) - \varphi_{offset\_x} + \varphi_{noise\_x} \left( {idx, mdy, 0} \right)} \right\} + \mathop \sum \limits_{j = 0}^{m - 1} \left\{ {\Delta \varphi_{oy} \left( {0, jdy, 0} \right) - \varphi_{offset\_y} + \varphi_{noise\_y} \left( {0, jdy, 0} \right)} \right\} $$

7 $$ \varphi \left( {ndx, mdy, 0} \right)_{M} = \varphi_{0} + \mathop \sum \limits_{i = 0}^{n - 1} \left\{ {\Delta \varphi_{ox} \left( {idx, 0, 0} \right) - \varphi_{offset\_x} + \varphi_{noise\_x} \left( {idx, 0, 0} \right)} \right\} + \mathop \sum \limits_{j = 0}^{m - 1} \left\{ {\Delta \varphi_{oy} \left( {ndx, jdy, 0} \right) - \varphi_{offset\_y} + \varphi_{noise\_y} \left( {ndx, jdy, 0} \right)} \right\} $$

Suppose that the phase noise is random noise, the standard deviation for the averaged data can be calculated as $\sigma = \frac{{\sqrt {\sigma_{Npass}^{2} + \sigma_{Mpass}^{2} } }}{2}$. When $\sigma_{Npass}^{2} = \sigma_{Mpass}^{2}$, the resultant standard deviation will be $\sigma = \frac{{\sqrt {2\sigma_{Npass}^{2} } }}{2} = \frac{{\sigma_{Npass} }}{\sqrt 2 }$. Note that the spatial resolution is limited by the spatial separation of the probes. If dx, dy, and dz are less than λ/2, where λ is the wavelength of the electric field to be measured, the phase distribution can be interpolated. Note that the assemble accuracy of the integrated multichannel probe is discussed in Supplementary (see Fig. S2). To compensate for mechanical misalignments, we use measured values of dx, dy, and dz, which are dx = 1.24 mm, dy = 1.42 mm, and dz = 0.625 mm, respectively.

## Method

**Figure 3:**
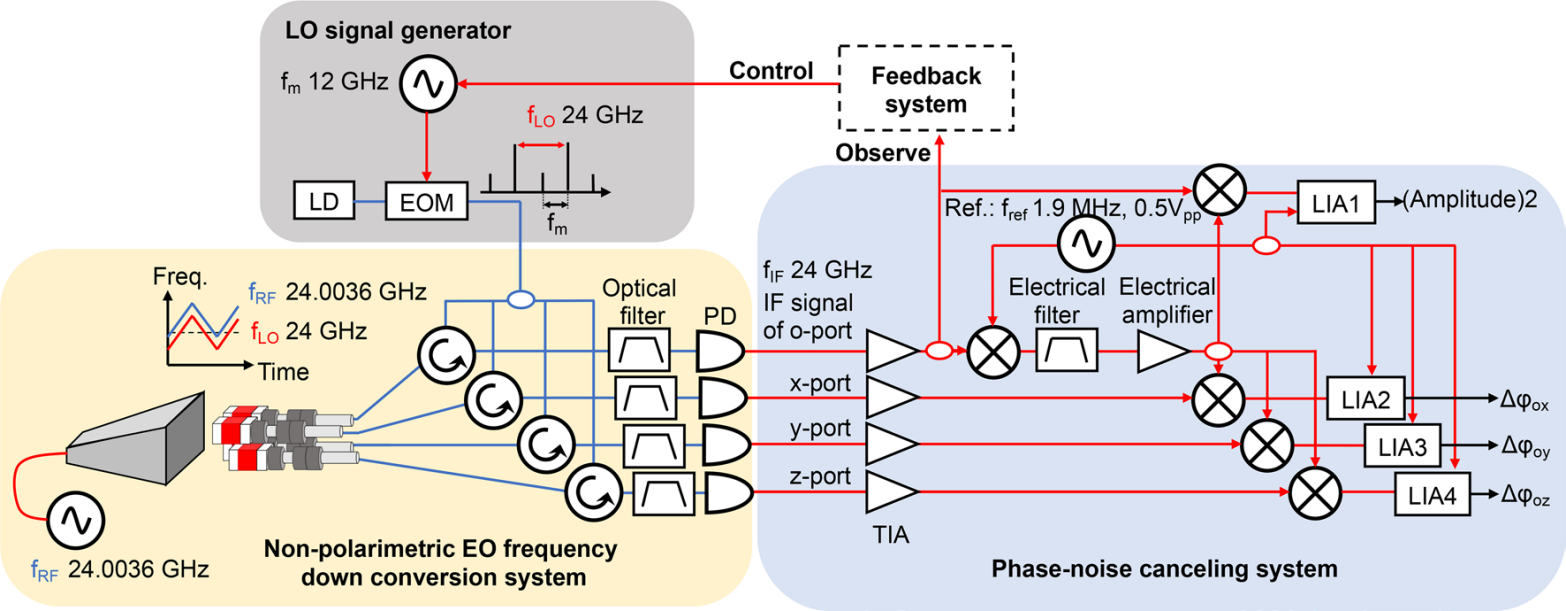
Setup for proof-of-concept experiments using integrated multichannel probe. Blue lines are optical fibers while red lines are electrical cables. *LO* local oscillator, *EOM* electro-optic modulator, *LD* laser diode, *PD* photodiode, *LIA* lock-in amplifier.

Figure 3 shows the setup for the proof-of-concept experiment. An FMCW signal with a center frequency of 24.0036 GHz and a frequency deviation of 40 MHz is generated by a synthesizer. The repetition period of the FMCW signal is 2.5 ms. Note that the deviation and repetition period are limited by the synthesizer used. The antenna feeding power is approximately 24 dBm. The photonic local oscillator (LO) signal is generated by the EO modulator. The center frequency of the signal to be measured is 24.0036 GHz; therefore, the frequency of the modulation signal is set to be 12 GHz and is supplied by a local synthesizer. The quasi two-tone optical signal is launched to each EO sensor of the integrated multichannel probe through the optical power divider and circulators. The frequency down-conversion is based on the nonpolarimetric technique^33^, and the detected intermediate frequency (IF) signal (3.6 MHz) is monitored and fed back to the local synthesizer to track the frequency of the FMCW signal. The IF signal detected by port O is electrically up-converted by mixing with the reference signal. The frequency and amplitude of the reference signal are 1.9 MHz and 0.5 V_pp_, respectively. The up-converted signal is filtered and amplified, and then, mixed with four signals: the IF signals from ports O, X, Y, and Z. The mixed signals are detected by the lock-in amplifiers to measure the amplitude and phase differences between port O and each remaining port. In this measurement scheme, the local signals for the LO signal generation and electrical up-conversion are not phase-locked to the FMCW signal to be measured. Frequency tracking reduces the bandwidth of the noise-cancelling electronics, and hence, improves the SNR. The frequency of the photonic LO can easily be extended to the millimeter-wave band, by increasing the modulation power or modulation frequency. Note that the effect of the disturbance that may be caused by the integrated multichannel probe on the accuracy of the measurements is discussed in Supplementary (see Fig. S3 and Table S1).

## Results and Discussion

### Variation of the measurement using integrated multichannel probe with proposed scheme

**Figure 4:**
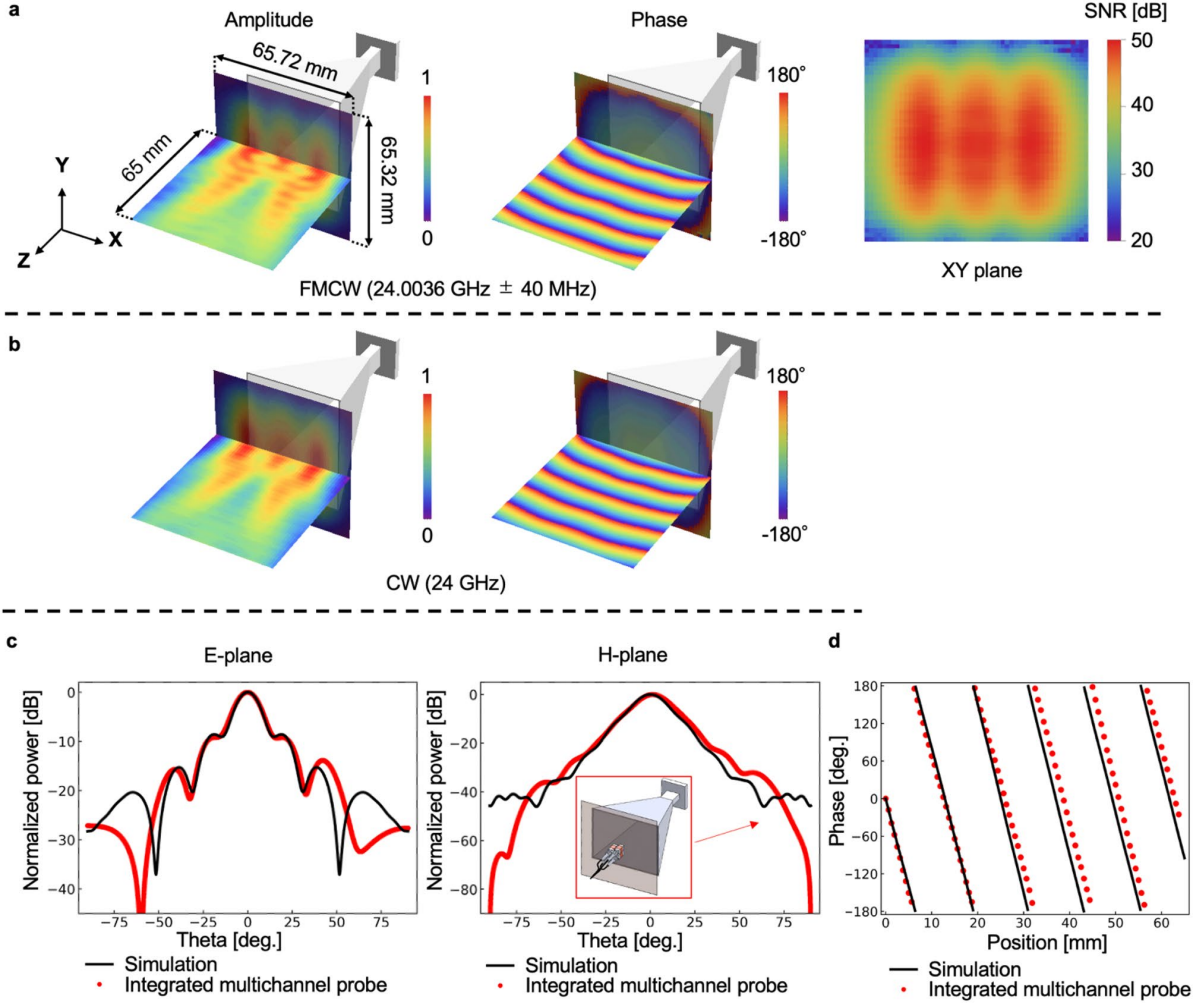
Near-field measurements and far-field characterization of a horn antenna. (**a**) Visualized amplitude and phase distributions of the 24 GHz ± 40 MHz frequency-modulated continuous wave (FMCW) signal radiated from the horn antenna. (**b**) Simulation results for 24 GHz continuous wave (CW) signal. (**c**) Radiation pattern calculated from the near-field distribution. The black line represents the simulation results for the 24 GHz CW signal. (**d**) Measurement results of phase development in the Z-axis direction. The black line is the result of the simulation.

To validate the proposed measurement scheme, we demonstrate the FMCW signal visualization based on the asynchronous phase-difference measurement using the integrated multichannel probe with the configuration shown in Fig. 1c. In this proof-of-concept experiment, we measured the near-field of a standard horn antenna and compared the measured near-field distribution with a simulated one to verify the accuracy and fidelity of the measurements. Note that the simplest electromagnetic scenario is also chosen for this verification to eliminate the uncertainty in the simulation model. Figure 4 shows the amplitudes and phase distributions of the measured (Fig. 4a) and simulated (Fig. 4b) results. The simulation was conducted for a CW signal at a frequency of 24 GHz using antenna model shown in Fig. S4 (Supplementary). Note that the frequency deviation of ± 40 MHz relative to the carrier frequency of 24 GHz is ± 0.17%, which is small enough not to change the far-field pattern. In Fig. 4, the horn antennas are the simulation models, that is, the results of Fig. 4a are obtained by merging the simulation model and the experimental results. The measurement area of ​​the XY plane is 65.72 mm × 65.32 mm, which is sufficient for far-field calculation. As it can be observed, the measured amplitude and phase distributions agree well with the simulated distributions. The radiation patterns calculated from the measured 2D near-field distributions are also shown in Fig. 4c. The black lines and red dots represent the simulated and measured results, respectively. As shown in the figure, the overall characteristics agree well with the simulated results. Figure 4d shows the 1D phase distribution in the Z-axis direction. The black line and red dots are the simulated and measured results, respectively. The gradients of the phase development in the Z-axis are calculated by the least-squares fitting method. The measured gradient is − 28.2°/mm, and that obtained by the simulation is − 28.8°/mm, which are in good agreement. Note that the wavelength of the 24 GHz wave is 12.5 mm; therefore, the wave number is calculated as k = 2π/λ = 28.8°/mm.

Table 1 summarizes the side-lobe power relative to the main lobe for the E-plane and 3 dB beam widths for the E- and H- planes. In the case of the 3 dB beam width and the position and power of the first sidelobe, the results obtained by the proposed system agree well with the simulation results. However, there are discrepancies between the measurement and simulation, in terms of the position of the second sidelobe. The discrepancy is approximately 1.40 dB and 3.4° for the power and position, respectively, and is significant when compared with the results measured by the conventional system (1.17 dB and 1.2°, in Table 1). This discrepancy can be attributed to the phase-difference measurement and not to the integrated multichannel probe. One reason for this discrepancy could be the phase-noise accumulation during phase retrieval. This accumulated phase noise masks small undulations in the phase distributions, which degrade the accuracy at higher spatial-frequency components. Averaging multiple paths, rather than the two paths of N and M as shown in Fig. 2d, during phase retrieval, would reduce the phase noise.

**Table 1:** Characteristics of radiation pattern.

	Integrated multichannel probe w/o reference probe	Simulation	Discrepancy between measurement and simulation
H-plane 3 dB beam width (°)	17.2	15.9	1.3
E-plane 3 dB beam width (°)	14.8	13.9	0.9
**E-plane**			
First side-lobe			
Position (°)	19.5	18.9	0.6
Main lobe ratio (dB)	− 9.22	− 8.59	0.63
Second side- lobe			
Position (°)	42.0	38.6	3.4
Main lobe ratio (dB)	− 13.87	− 15.27	1.40

The measurement is conducted for a 24 GHz ± 40 MHz frequency-modulated continuous wave (FMCW) signal, using the configuration shown in Fig. 1c.

### Far-field characterization of the car-bumper-transmitted FMCW radar signal

**Figure 5:**
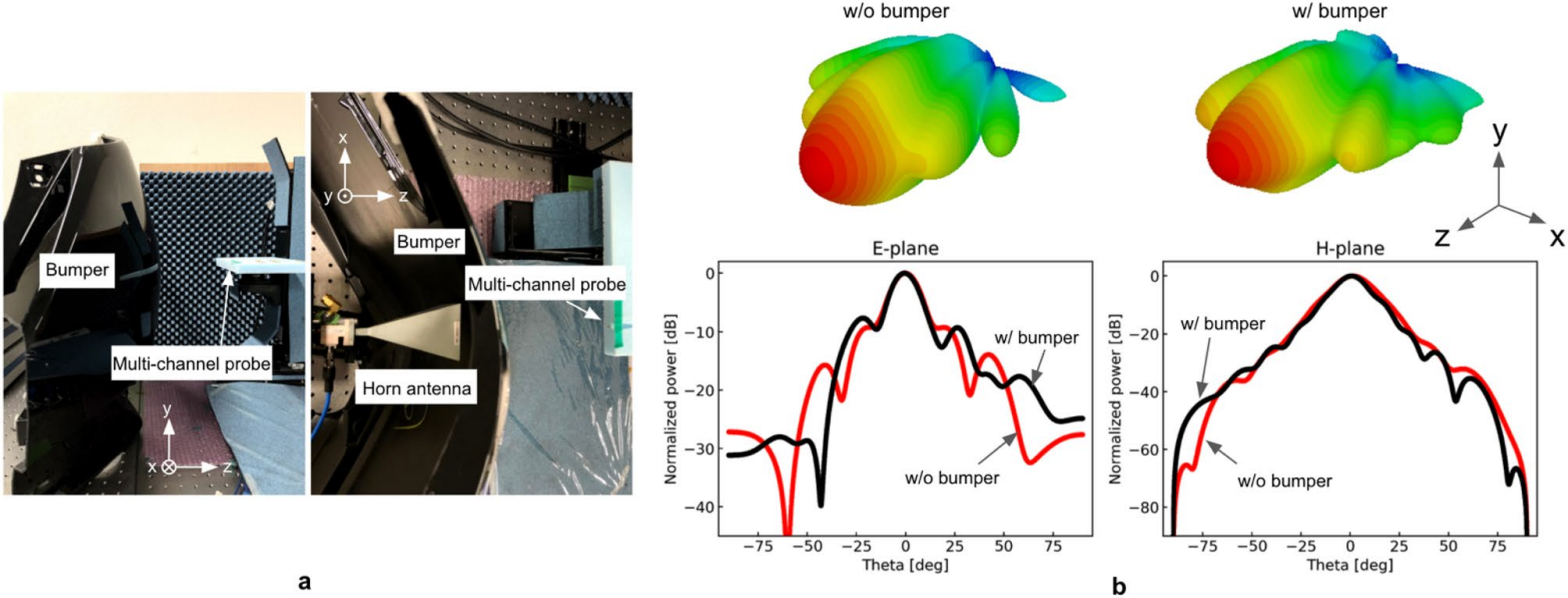
Far-field characterization of the horn antenna with and without a car bumper. (**a**) Experimental situation. A car bumper is installed just in front of the horn antenna. (**b**) 1D and 2D radiation patterns with and without the bumper.

We place a car bumper just in front of the horn antenna, which is characterized in the former sections and measure the near-field using the integrated multichannel probe to compare the far-field distribution with and without the bumper. The experimental situation is depicted in Fig. 5a. The experiment imitates a realistic situation of a bumper installed vehicle in which the reference probe cannot be placed in the source side**;** therefore, the conventional technique is not applicable. In practice, we could not find a location to place the fixed reference probe where it does not mechanically interfere with the measurement probe and also detects sufficient SNR for the measurement. The FMCW signal was radiated, and the field distribution just after the bumper was measured with the multichannel probe using the setup shown in Fig. 3. The 2D and 1D far-field patterns with and without the bumper are shown in Fig. 5b. In particular, there was a change in the positions and levels of the side-lobes. The radiation pattern was altered by the bumper installation, especially in the E-plane. The asymmetric far-field distribution in the E-plane is due to the asymmetric curvature of the bumper shown in Fig. 5a. The FWHM of the beam in the E-plane changed slightly from 14.8°to 14.2°. This discrepancy is smaller than that between the simulation and measurement results without the bumper (see Table 1). On the other hand, the first side-lobe to main lobe ratio in the E-plane degraded from -9.2 dB to -7.6 dB. The degradation of 1.6 dB is larger than the discrepancy between the simulation and measurement results without the bumper, while it might have an impact on radar detection performance.

## Conclusion

We proposed a new asynchronous measurement technique using an integrated multichannel probe to visualize the spatial distribution of the electric near-field and calculate a far-field pattern, as an alternative to the previous VNA-based measurement. The system required neither a reference probe nor a cable plugged to the RF source, to acquire or inject the reference signal for phase measurements. To validate the proposed approach, we visualized the near-field distribution of the FMCW signal (center frequency: 24 GHz, frequency deviation: ± 40 MHz, repetition period: 2.5 ms) and calculated a far-field pattern from the measured near-field. The average discrepancy between the measurement and simulation (24 GHz CW signal) for the 3 dB beam width and first side-lobe position were approximately 1.1° and 0.6°, respectively. The simulated power of the first side-lobe, relative to the main lobe, was − 8.59 dB, whereas the calculated value from our measurement was − 9.22 dB, which indicated a discrepancy of less than 1 dB. As a proof-of-concept, we demonstrated that the proposed technique can reveal the radiation pattern degradation of the FMCW signal due to a car bumper installation. The experiment imitates a realistic situation of a bumper installed vehicle in which the reference probe cannot be placed on the source side or the RF signal cannot be supplied to the source. Significant differences exist among with and without the bumper installation, especially in the levels and the positions of the side-lobes in the E-plane. The results show that the proposed technique paved the way for the car radar inspection in real situations. Owing to photonics, our technique introduced a negligible disturbance in the field to be measured. The system could be applied to another frequency band by simply changing the modulation frequency and/or power of the synthesizer for photonic LO generation. In principle, the photonic LO tracks any type of frequency/phase-modulated signal. Moreover, our new scheme can be used to investigate antennas with complex modes and polarization^34^, through the polarization state measurement method demonstrated in^35^. Although there is still some room for improving the measurement accuracy, particularly, for the higher spatial-frequency components, the asynchronous measurement technique using the proposed integrated multichannel probe would be a promising and versatile inspection technique for on-chip antenna devices, in-vehicle radars, etc.

## Supplementary information

Supplementary information.
