# Supplementary material for: Asynchronous electric field visualization using an integrated multichannel electro-optic probe
Source: Sci Rep. 2020 Oct 5;10:16479. doi: 10.1038/s41598-020-73538-7 (PMC7536238; doi:10.1038/s41598-020-73538-7)
Supplement: Supplementary file 1 — Supplementary information. [file 41598_2020_73538_MOESM1_ESM.docx]

**[Supplementary]**

**Asynchronous electric field visualization using an integrated**

**multichannel electro-optic probe**

Shintaro Hisatake^1^, Junpei Kamada^1^, Yuya Asano^1^, Hirohisa Uchida^2^, Makoto Tojo^3^, Yoichi Oikawa^3^, and Kunio Miyaji^3^

^1^Department of Electrical, Electronic and Computer Engineering, Gifu University, Gifu 501-1193, Japan

^2^ Arkray Inc., Kyoto 602-0008, Japan

^3^Think-Lands Co., Ltd., Yokohama 230-0046, Japan

**Electrooptic probe**

Fig. S1 shows the construction of the single-channel electrooptic (EO) probe. The probe consists of a high-reflective (HR)-coated BK7, DAST crystal, spacer (BK7), and graded-index (GRIN) lens attached to the polarization-maintaining (PM) optical fiber. The polarization direction of the optical signal is aligned to the slow-axis of the PM fiber. The minimum detectable field strength of the DAST probe used in the experiments was estimated to be approximately 0.3V/m.

**Fig. S1.** **Schematic of the single-channel EO probe.** It consists of an HR coated BK7, DAST crystal, and GRIN lens.

The principle of field detection is based on the phase modulation in the DAST crystal. Therefore, our EO probe is considerably less sensitive to temperature fluctuations compared with the polarization modulation type EO probe, in which the natural birefringence can fluctuate with temperature. We have evaluated the effect of fiber movement in a previous study [S1]. The results guarantee that there exists no sensitivity fluctuations and additive noises caused by fiber movement.

**
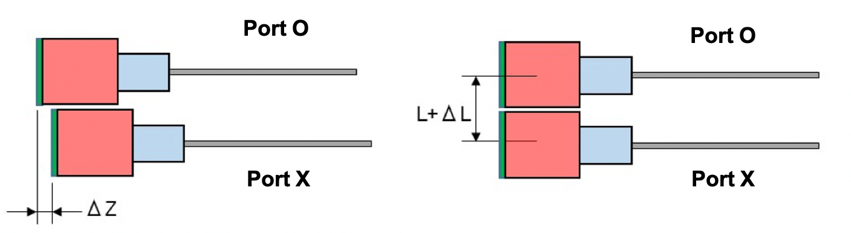
**

**Fig. S2 | Assembly accuracy of the integrated multichannel probe.**

The multichannel probe consists of four single-channel probes. The mechanical misalignment of the ΔZ and ΔL shown in Fig. S2 were less than 0.1 μm and 20 μm, respectively. These values were quite small compared to the wavelength of the millimeter-wave; therefore, the impact of the misalignment will be negligible. In the electric field measurements, we employed the measured dx, dy, and dz values.

**Evaluation of integrated multichannel probe**

The EO probe consists of dielectric materials; it does not contain any metallic components; therefore, the disturbance to the field is drastically reduced compared with the open-ended waveguide [S2]. The EO probe is invisible to the near-field and this is one of the advantages of EO measurement. Thus far, previous studies have evaluated single-channel probes [S2, S3]; therefore, here we examine the effect of the disturbance that may be caused by the integrated multichannel probe on the accuracy of the measurements. A 24 GHz continuous wave (CW) is radiated from a standard horn antenna, and the 2D distributions of the near-field at the antenna surface are measured using the conventional configuration shown in Fig. 1b. We compare the results obtained by port O of the integrated multichannel probe used as a single-channel probe and those obtained using an actual single-channel probe. In this comparison, the measurement system was based on the conventional scheme for both experiments to eliminate any differences that may be caused by the new measurement scheme. Note that another single-channel probe set in a shielding box (not shown in Fig. 1b) is used for the reference-signal measurements.

The disturbance caused by the probe alters the far-field pattern. Therefore, we quantitatively compare the far-field pattern to evaluate the accuracy of the measurements. Figure S3 shows the far-field patterns calculated from the measured amplitude and phase distributions of the near-field at Z = 9.0 mm (0.72λ). The radiation power was normalized to its maximum. In the figure, the red and blue dots are the results obtained by using port O of the integrated multichannel probe and the single-channel probe, respectively. The black lines are the results of the simulation (CST Studio Suite). The obtained results agree well with each other for both E- and H- planes, indicating that the disturbance caused by the integrated multichannel probe is as small as that caused by the single-channel probe.

**Fig. S3 | Far-field patterns calculated from measured near-field distributions for CW signal, using single-channel probe and port O of the integrated multichannel probe.** Black lines show the simulation results. The red and blue dots are the results obtained using port O of the integrated multichannel probe and the single-channel probe, respectively, with the configuration shown in Fig. 1b.

Table S1 summarizes the sidelobe power relative to the main lobe for the E-plane and the 3 dB beam widths for the E- and H- planes. In the case of the 3 dB beam widths, the results measured by the single-channel probe, port O of the integrated multichannel probe, and simulations agree very well with each other. The difference is within 0.3°. The position and relative power of the first side lobe also agree well and are within 2.1° and 0.57 dB, respectively.

**Table S1 | Characteristics of radiation pattern.** The measurements are conducted for a 24 GHz continuous wave signal with the configuration shown in Fig. 1b.

|  | | | Integrated multichannel probe w/ reference probe | Signal-channel probe w/ reference probe | Simulation |
| --- | --- | --- | --- | --- | --- |
| H-plane 3dB beam width [deg.] | | | 15.7 | 15.9 | 15.9 |
| E-plane 3dB beam width [deg.] | | | 13.9 | 13.6 | 13.9 |
| E-plane | first side-lobe | Position [deg.] | 17.2 | 19.3 | 18.9 |
|  |  | Main lobe ratio [dB] | -9.13 | -9.16 | -8.59 |
|  | second side- lobe | Position [deg.] | 39.8 | 40.4 | 38.6 |
|  |  | Main lobe ratio [dB] | -16.44 | -15.87 | -15.27 |

**Calculations**

The simulations were conducted using CST Studio Suite software. The calculations were based on a finite integration technique. In order to accelerate the calculation, simulations were performed using a GPU. The CPU of the machine used was Intel (R) Core (TM) i7-7700K, the GPU was NVIDIA Quadro P6000, and the memory size was 64 GB. As shown in Fig. S4, the range of 70 mm in the x-axis direction, 70 mm in the y-axis direction, and 77.5 mm in the z-axis direction was divided into 7933210 meshes and analyzed using the time domain solver. The analysis took 142 s for the near-field calculation.

**Fig. S4.** **Model for the simulations.**

As for the far-field calculation, we imported measured near-field data (amplitude and phase) into the CST simulator as a field source, and propagation was calculated based on the time-domain solver.

**Reference**

1. Hisatake, S. et al. Visualization of the spatial–temporal evolution of continuous electromagnetic waves in the terahertz range based on photonics technology. *Optica* volume 1, 365-371 (2014)
2. Lee, D.-J. & Whitaker, J. F. An optical-fiber-scale electro-optic probe for minimally invasive high-frequency field sensing. *Opt. Express* 16, 21587–21597 (2008).
3. Hisatake, S. et al. Mapping of electromagnetic waves generated by free-running self-oscillating devices. *Sci. Rep.,* volume **7,** article number 9203 (2017)
